# Comparison of seven serum assays on four automatic analysers

**DOI:** 10.1155/S1463924689000271

**Published:** 1989

**Authors:** Lucia Sacchetti, Dionigio Cerasuolo, Marco Besozzi, Antonio D'Agostino, Carlo Franzini, Francesco Salvatore

**Affiliations:** 1 Dipartimento di Biochimica e Biotecnologie Mediche Universitià di Napoli Via S. Pansini 5 Napoli 80131 Italy; 2 Laboratorio di Analisi Ospedale Filippo del Ponte Via F. del Ponte 19 Varese 21100 Italy

## Abstract

Serum enzyme assays used on four different analysers (Hitachi 737, Hitachi 705, Cobas-bio and RA-2X) were compared by determining the activity of seven different enzymes (AST, ALT, LD, ALP, GGT, CK and AMS). Performance checks (quality control procedure) and replications (the study of the total analytic imprecision and of its components) were conducted and the methods were compared by linear regression analysis with statistical inference on the curves following the protocols of the National Committee for Clinical Laboratory Standards (NCCLS: PSEP- 2, PSEP-3, PSEP-4). The correlation coefficients between the methods (r = 0.991-0.999), together with the other statistical parameters, indicated that the methods are well correlated on all the instruments. The total imprecision was good for all analytes, except ALT. Among the instruments tested, the RA-2X gave more variable results, although the total imprecision was acceptable.

There was no relevant carry-over effect. The evaluation of performance claims indicated that the expected error did not substantially affect the results at the level of clinical decisions.


Journal of Automatic Chemistry Vol. 11, No. 3 (May–June 1989), pp. 124–128

### Comparison of seven serum assays on four automatic analysers

**Lucia Sacchetti[1], Dionigio Cerasuolo[1], Marco Besozzi[2], Antonio D'Agostino[1], Carlo Franzini[2] and Francesco Salvatore[1]***

*[1]Dipartimento di Biochimica e Biotecnologie Mediche, Università di Napoli, Via S. Pansini 5, 80131 Napoli, Italy, and [2]Laboratorio di Analisi, Ospedale Filippo del Ponte, Via F. del Ponte 19, 21100 Varese, Italy*

*Serum enzyme assays used on four different analysers (Hitachi 737, Hitachi 705, Cobas-bio and RA-2X) were compared by determining the activity of seven different enzymes (AST, ALT, LD, ALP, GGT, CK and AMS). Performance checks (quality control procedure) and replications (the study of the total analytic imprecision and of its components) were conducted and the methods were compared by linear regression analysis with statistical inference on the curves following the protocols of the National Committee for Clinical Laboratory Standards (NCCLS: PSEP-2, PSEP-3, PSEP-4). The correlation coefficients between the methods (r = 0.991–0.999), together with the other statistical parameters, indicated that the methods are well correlated on all the instruments. The total imprecision was good for all analytes, except ALT. Among the instruments tested, the RA-2X gave more variable results, although the total imprecision was acceptable. There was no relevant carry-over effect. The evaluation of performance claims indicated that the expected error did not substantially affect the results at the level of clinical decisions.*

### Introduction

Enzyme assays are widely used and play an important role in laboratory medicine, hence the need for a detailed analysis of the analytical error and of its various components, also in relation to the various methods and instruments that are commercially available and the need for the commutability of results among various laboratories and often even within the same institution. The most effective method for this type of evaluation remains the analysis of replicates and the comparison between methods [1]. Therefore, we studied the analytical error for seven serum enzymatic activities that are widely used in clinical diagnostics [AST (aspartate aminotransferase), ALT (alanine aminotransferase), LD (lactate dehydrogenase), ALP (alkaline phosphatase), GGT (gamma-glutamyl transferase), CK (creatine kinase) and AMS

*Table 1. Imprecision (carry-over included) for the Hitachi 737 ($H_1$), Hitachi 705 ($H_2$), Cobas-Bio (C) and RA-2X (R) (each enzyme activity was measured at three different levels: low, medium and high).*

| | | | | | Imprecision (CV, %) | | | | | | | | | | | | | | | |
|---|---|---|---|---|---|---|---|---|---|---|---|---|---|---|---|---|---|---|---|---|
| Analyte | Mean (U/l) | | | | Within-run | | | | Between runs, within-day | | | | Between days | | | | Total | | | |
| | $H_1$ | $H_2$ | C | R | $H_1$ | $H_2$ | C | R | $H_1$ | $H_2$ | C | R | $H_1$ | $H_2$ | C | R | $H_1$ | $H_2$ | C | R |
| AST | 14 | 17 | 16 | 16 | 3.8 | 3.6 | 4.6 | 8.8 | 4.2 | 2.8 | 4.1 | 8.2 | 4.7 | 4.5 | 5.2 | 9.9 | 6.1 | 5.8 | 6.9 | 13.3 |
| | 27 | 31 | 30 | 30 | 2.0 | 1.8 | 2.5 | 6.7 | 2.1 | 2.0 | 2.5 | 4.1 | 2.7 | 3.3 | 3.2 | 8.8 | 3.4 | 3.8 | 4.0 | 11.1 |
| | 176 | 190 | 196 | 167 | 0.6 | 0.8 | 1.1 | 1.8 | 1.9 | 1.9 | 1.9 | 2.9 | 3.3 | 3.4 | 3.9 | 5.4 | 3.4 | 3.5 | 4.0 | 5.7 |
| ALT | 9 | 11 | 13 | 8 | 7.2 | 6.6 | 8.5 | 20.3 | 6.8 | 6.8 | 6.5 | 10.2 | 9.6 | 10.1 | 9.8 | 18.4 | 12.0 | 12.0 | 12.9 | 27.4 |
| | 14 | 16 | 16 | 13 | 5.2 | 4.5 | 5.9 | 11.2 | 5.6 | 6.7 | 9.7 | 8.6 | 13.4 | 11.6 | 11.0 | 19.2 | 14.4 | 12.4 | 12.4 | 22.2 |
| | 103 | 109 | 108 | 94 | 0.8 | 0.6 | 1.2 | 3.3 | 3.6 | 4.2 | 3.6 | 5.4 | 10.3 | 11.1 | 12.7 | 13.2 | 10.4 | 11.2 | 12.8 | 13.6 |
| LD | 172 | 164 | 168 | 84 | 1.3 | 1.5 | 3.1 | 6.6 | 1.6 | 1.1 | 1.8 | 5.9 | 3.1 | 2.3 | 2.1 | 8.4 | 3.3 | 2.7 | 3.7 | 10.7 |
| | 310 | 298 | 304 | 149 | 1.2 | 3.9 | 1.6 | 1.8 | 1.2 | 1.5 | 1.5 | 2.3 | 2.2 | 2.2 | 2.0 | 5.7 | 2.5 | 4.5 | 2.6 | 5.9 |
| | 729 | 688 | 739 | 339 | 1.0 | 0.6 | 2.4 | 1.6 | 0.9 | 1.1 | 2.1 | 2.3 | 2.9 | 1.8 | 3.0 | 3.4 | 3.0 | 1.9 | 3.9 | 3.8 |
| ALP | 101 | 106 | 104 | 103 | 1.8 | 0.6 | 1.6 | 2.5 | 3.6 | 1.2 | 1.6 | 2.1 | 6.1 | 3.1 | 2.7 | 3.0 | 6.4 | 3.2 | 3.2 | 3.9 |
| | 199 | 206 | 202 | 202 | 1.5 | 0.8 | 1.5 | 2.3 | 1.8 | 1.2 | 1.8 | 1.5 | 5.4 | 3.0 | 2.7 | 3.2 | 5.6 | 3.1 | 3.1 | 4.0 |
| | 575 | 600 | 592 | 589 | 1.5 | 0.5 | 1.1 | 1.4 | 2.9 | 2.1 | 1.1 | 1.9 | 5.8 | 2.9 | 2.3 | 3.3 | 6.0 | 3.0 | 2.5 | 3.6 |
| GGT | 20 | 19 | 20 | 20 | 4.4 | 3.1 | 2.7 | 4.6 | 2.1 | 2.3 | 1.9 | 2.7 | 2.8 | 4.7 | 2.9 | 4.4 | 5.2 | 5.6 | 3.9 | 6.4 |
| | 30 | 31 | 31 | 31 | 5.8 | 2.1 | 1.8 | 7.6 | 1.9 | 1.6 | 1.5 | 3.3 | 2.6 | 3.6 | 3.3 | 4.2 | 6.3 | 4.1 | 3.8 | 8.7 |
| | 194 | 200 | 197 | 216 | 0.9 | 0.7 | 1.1 | 1.4 | 0.9 | 1.0 | 0.6 | 1.8 | 1.5 | 1.2 | 1.4 | 3.3 | 1.8 | 1.4 | 2.0 | 3.6 |
| CK | 64 | 59 | 58 | 58 | 3.5 | 2.3 | 4.1 | 9.2 | 5.4 | 1.8 | 3.3 | 3.4 | 6.0 | 3.5 | 1.7 | 3.0 | 6.9 | 4.2 | 4.4 | 9.7 |
| | 104 | 101 | 98 | 98 | 1.6 | 2.1 | 4.1 | 3.3 | 4.3 | 2.1 | 2.3 | 2.1 | 5.2 | 2.7 | 3.0 | 4.9 | 5.4 | 3.5 | 5.1 | 5.9 |
| | 700 | 707 | 691 | 640 | 0.9 | 0.5 | 1.6 | 5.4 | 1.1 | 1.6 | 1.2 | 2.2 | 3.0 | 2.4 | 2.2 | 4.9 | 3.1 | 2.5 | 2.7 | 7.3 |
| AMS | 72 | 69 | 72 | 39 | 5.3 | 2.8 | 1.6 | 8.9 | 3.6 | 3.2 | 2.1 | 4.3 | 1.7 | 3.7 | 2.6 | 10.1 | 5.6 | 4.7 | 3.0 | 13.4 |
| | 138 | 132 | 135 | 82 | 5.5 | 1.5 | 1.2 | 11.1 | 5.3 | 1.1 | 2.3 | 5.8 | 2.5 | 2.5 | 1.6 | 7.3 | 6.1 | 2.9 | 2.0 | 13.3 |
| | 874 | 860 | 864 | 628 | 0.9 | 0.6 | 1.6 | 1.1 | 1.2 | 1.0 | 0.8 | 2.0 | 1.6 | 1.7 | 1.4 | 2.2 | 1.8 | 1.8 | 2.1 | 2.4 |

* To whom correspondence should be addressed.

0142–0453/89 $3.00 © 1989 Taylor & Francis Ltd.

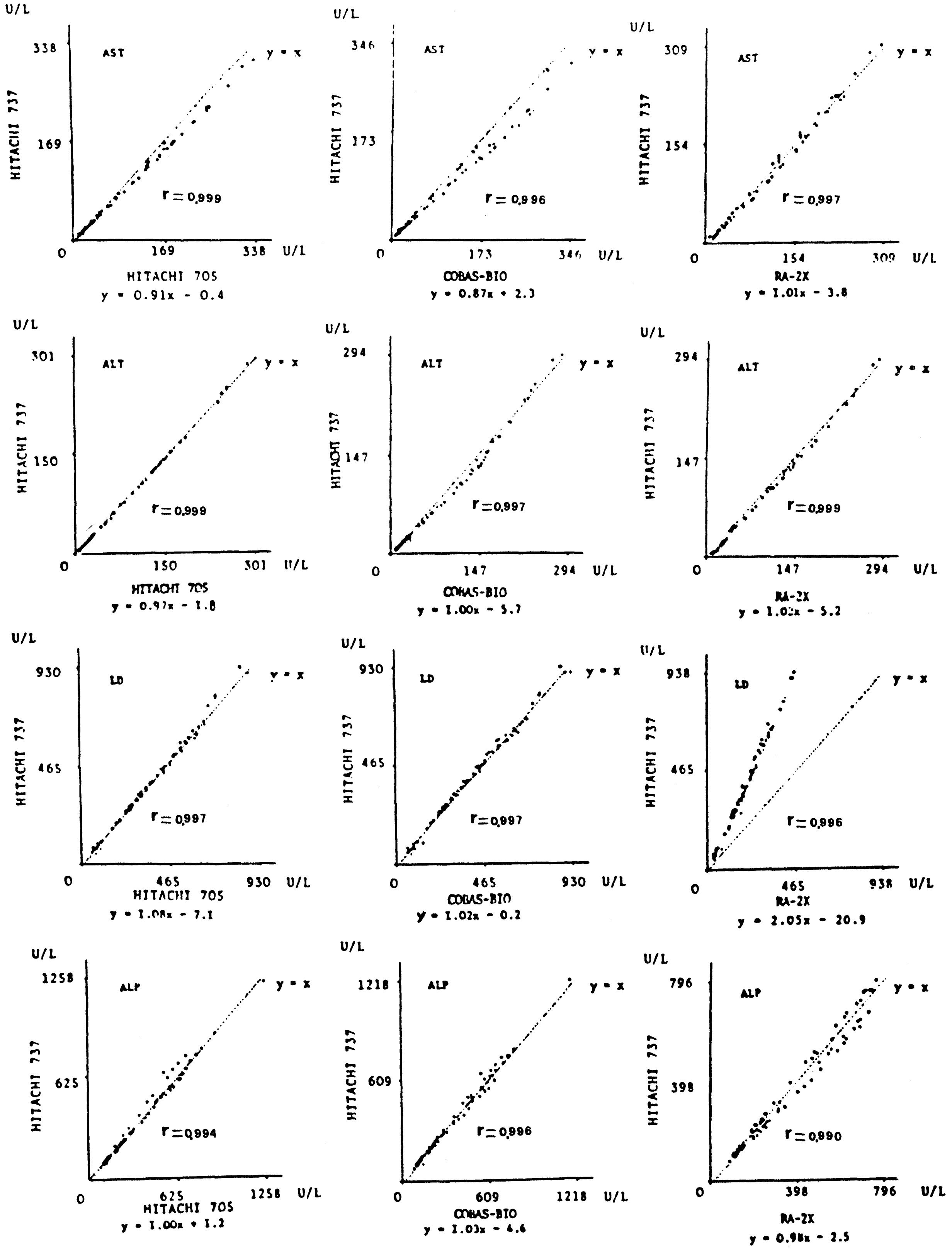

AST
r = 0,999
HITACHI 705
y = 0.91x - 0.4
AST
r = 0,996
COBAS-BIO
y = 0.87x + 2.3
AST
r = 0,997
RA-2X
y = 1.01x - 3.8
ALT
r = 0,999
ALT
r = 0,997
COBAS-BIO
y = 1.00x - 5.7
ALT
r = 0,999
RA-2X
LD
r = 0,997
HITACHI 705
LD
r = 0,997
COBAS-BIO
y = 1.02x - 0.2
LD
r = 0,996
RA-2X
y = 2.05x - 20.9
ALP
r = 0,994
HITACHI 705
y = 1.00x + 1.2
ALP
r = 0,996
COBAS-BIO
ALP
r = 0,990
RA-2X
HITACHI 737
U/L
y = x

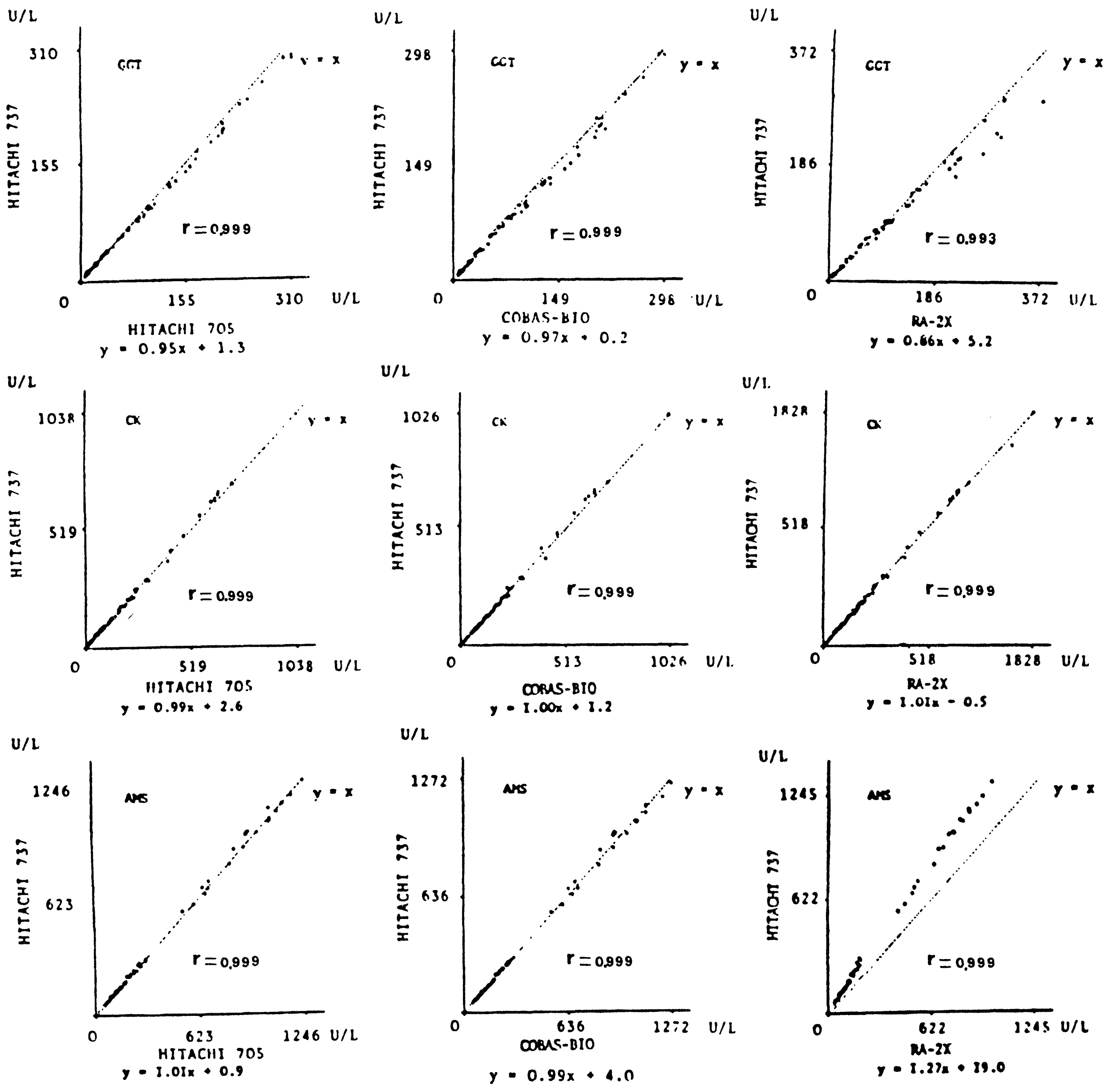

*Figure 1. Comparison between methods on the four instruments performed by linear regression analysis (n = 100) (least-squares method) and correlation (different analytical methods were used for LD, GGT and AMS assays on the RA-2X).*

(amylase)] measured with four instruments [Hitachi 737 and Hitachi 705 (Boehringer Biochemia Robin), Cobas-Bio (Roche) and RA-2X (Technicon Instruments)]. These instruments were selected because they are representative of at least three different approaches. Lastly, we also compared the Hitachi 737, which is one of the newest autoanalysers, and its procedures with the other instruments and their respective procedures.

### Experimental

### *Instruments*

The following were used: Hitachi 737 (Boehringer, Mannheim, FRG), a discrete, selective multi-channel analyser; Hitachi 705 (Boehringer), a discrete, selective multi-channel analyser; Cobas-Bio (Roche, Milan, Italy), a fast centrifugal analyser; and RA-2X (Technicon Instruments, Tarrytown, NY, USA), a discrete, selective multi-channel analyser in which an inert fluid (fluorocarbon) is used to prevent cross-contamination. The Pentalab (Poli, Milan, Italy) statistical software package was used.

### *Enzymatic assays*

All the catalytic activities were assayed at 37 °C. All the reagents were from Boehringer, except for those used with the RA-2X, which came from Technicon. The methods employed for AST, ALT, ALP, CK and LD were according to SCE (Scandinavian Committee on Enzymes) recommendations [2,3] on all the instruments, except for the RA-2X where the lactate→pyruvate reaction was used for LD determination [4]. GGT activity was determined according to Szasz and Persijn [5] except on the RA-2X, where SCE recommendations were also followed [6]. For the AMS assay, 4-NP-maltoheptaoside [7] was used as substrate on all instruments except the RA-2X, where maltotetraose was used [8].

*Replication experiment* [9]

Pools of human sera at three levels of concentration (low, medium and high) for each enzyme were frozen and used as required. Two analytical runs were performed each day (morning and afternoon) for 20 days; each run consisted of 18 replicate samples. This experiment was performed to obtain an estimate of the imprecision (total and of some components) of the test method.

*Comparison experiment* [10]

Fresh sera were obtained from patients ($n = 100$; 20% at or below the lowest 'Medical Decision Concentration,' but within a range of the lowest claimed measurement; 20% in the upper range of response of the measurement system claimed by the manufacturer; 20% above the expected 'normal' range at a 'Medical Decision Concentration;' 40% in the 'normal' range). Twenty-five samples were analysed twice each day, on all the instruments, within a maximum of 4 h. This experiment is the basis for making claims about inaccuracy.

*Performance check experiment* [11]

Commercial lyophilized control sera PN-U (mid-level concentration) (lot 746) and PP-U (high-level concentration) (lot 793) from Boehringer were used 1 h after reconstitution. Three aliquots of each control serum were included in each run of the comparison and of the replication experiments, in random order. The performance check is intended to ensure that the instrument performance is consistent with the expected performance during collection of the experimental data. If for any run the mean or range of the three mid- and high-level observations exceed the control limits (established prior to the experiment), the experimental data of that run should be discarded as suspected of not representing the typical performance of the method.

*Statistics*

The experimental data were elaborated using the Pentalab statistical software package, which is based on the statistical procedures recommended by NCCLS; it can be used on IBM personal and IBM compatible computers.

### Results

*Replication*

The results of this experiment are given in table 1. Variance analysis [9] was used to evaluate the total imprecision, and also the within-series, between-series, within-day and between-days components. The coefficients of variation (CV) are acceptable (<10%) for almost all methods tested on the Hitachi 705, Hitachi 737 and Cobas-Bio instruments; usually the highest CVs were obtained for low enzyme activity. ALT determination was affected by a greater variability (always <13%) because there was a loss of enzyme activity in serum pools during the experiment (storage: about 30 days at −20 °C). ALT variability was even greater on the RA-2X (CV 13–27%), on which instrument higher CVs (always <14%) were also usually obtained for all other methods.

*Comparison*

The values of the serum enzyme assays obtained with the Hitachi 737 were plotted against those obtained with the Hitachi 705, Cobas-Bio and RA-2X instruments, and were statistically assessed by linear regression analysis [10]. This statistical approach was used because the inherent measurement error of method $x$ is compensated for by the extended range of the data collected (see *Experimental*) and can therefore be ignored. The statistical significance of the regression equations was evaluated by means of the standard deviation from regression ($S_{yx}$), standard error of the slope ($S_b$) and of the intercept ($S_a$) (data not shown). Lastly, the correlation coefficients, $r$, were calculated ($r = 0.991$–$0.999$). The results, which are shown in figure 1, demonstrate that the methods are well correlated. The significantly different slopes obtained for LD and AMS on the RA-2X with respect to the other instruments tested were in agreement with the use on the RA-2X of different LD and AMS assay methods, each procedure having its own reference interval. This also explains the higher slope detected for GGT on the RA-2X, but in this instance the discrepancies fell within a range of values well above the upper reference limit and were therefore irrelevant for clinical purposes. For ALT and AST the use of the same calculation factor on Cobas-Bio led to a theoretical slope for ALT and to a higher slope for AST; why this happened is not clear, but in another experiment using a factor for AST different to that used for ALT we obtained results that were very satisfactory (data not shown).

*Performance check*

This test, performed prior to and during the comparison and the replicate experiments, ensures that the tested methods were in a stable state of operation. Optimum CVs for both PN-U and for PP-U (normal and pathological control sera) were obtained on all the instruments (CV <5%), except for the RA-2X where higher values were found (e.g., CV = 11.3% for ALT), in agreement with other reports [12]. The lower variability obtained in these experiments with respect to the replication experiment (reported in table 1) was obviously due to the different matrix and storage of the sera employed: in the first instance (performance check), commercial lyophilized control sera, reconstituted daily and used immediately; and in the second instance (replicate experiments), pooled sera from patients, collected and frozen, whose aliquots were defrozen and analysed each day. The numerical data obtained from the experiments described in this section are not reported for the sake of brevity.

*Carry-over*

Carry-over was evaluated according to the NCCLS procedure PSEP-3 [9]. Most of the carry-over values ($p$) are <1% (0.3–0.8% on the Hitachi 737; 0.3–0.6% on the Hitachi 705; 0.4–1.0% on the Cobas-Bio; and 0.6–1.4% on the RA-2X).

*Statement of performance claim*

The regression line was also used to calculate and verify the performance claim for the Hitachi 737 (method plus

*Table 2. Performance claims of the Hitachi 737 analyser (y method) versus the Hitachi 705 ($H_2$), the Cobas-Bio (C) and the RA-2X (R) analysers (x methods).*

| Analyte | Instrument | $X_c$ value* (U/l) | Tolerance limits Lower (U/l) | Upper (U/l) | Total error (U/l) |
|---|---|---|---|---|---|
| AST | $H_2$ | 60 | 48 | 60 | 12 |
| | C | 60 | 43 | 71 | 17 |
| | R | 60 | 49 | 73 | 13 |
| ALT | $H_2$ | 60 | 52 | 60 | 8 |
| | C | 60 | 41 | 67 | 19 |
| | R | 60 | 54 | 68 | 8 |
| LD | $H_2$ | 300 | 282 | 348 | 48 |
| | C | 300 | 272 | 342 | 42 |
| | R† | 150 | 242 | 330 | 180 |
| ALP | $H_2$ | 350 | 294 | 406 | 56 |
| | C | 350 | 311 | 401 | 51 |
| | R | 350 | 278 | 406 | 64 |
| GGT | $H_2$ | 50 | 43 | 55 | 7 |
| | C | 50 | 38 | 58 | 12 |
| | R† | 50 | 27 | 69 | 23 |
| CK | $H_2$ | 100 | 85 | 117 | 17 |
| | C | 100 | 82 | 119 | 19 |
| | R | 100 | 81 | 119 | 19 |
| AMS | $H_2$ | 240 | 211 | 275 | 35 |
| | C | 240 | 208 | 276 | 36 |
| | R† | 130 | 152 | 216 | 86 |

* $X_c$ = Medical Decision Concentration closest to the mean of the data used for method comparison.
† Assayed with methods different from those used on the other instruments.

instrument). This test serves to evaluate the inaccuracy from the estimate of bias at various Medical Decision Concentrations [13] and to evaluate inaccuracy plus imprecision (total error) at the Medical Decision Concentration, $X_c$, closest to the mean of the data used for method comparison. To estimate the total error we calculated, according to the NCCLS PSEP-4 procedure [10], the tolerance limits, i.e. the range which will (with 95% probability) contain the values of the $y$ method in 99% of all similar experiments. The results are given in table 2. The data indicate that, even under unfavourable experimental conditions, the expected value with the Hitachi 737 does not cause substantial alterations in the clinical decision levels with respect to the other methods.

### Discussion and conclusions

The random error evaluated by means of the imprecision study was similar to those already reported for the individual instruments for the same analytes [12, 14–16]. The imprecision increased at the lower activity levels for all the enzymes, as already reported by Schwartz *et al.* [12] for the RA-1000. The Hitachi 737, Hitachi 705 and Cobas-Bio showed a similar imprecision; a higher imprecision level was obtained for the RA-2X, but the value was comparable to data reported [12] for the same type of instrument. The low CVs (<5%) revealed by the performance check (replicate determinations of control sera) confirm the reliability of these instruments for the assay of the enzymatic activities tested. This finding is particularly interesting for the last generation instrument, the Hitachi 737, which even with random access and a greater throughput, produces results that are as precise as those obtained on the earlier instruments. The low carry-over values obtained in this study indicate that the instruments tested are suitable for use in clinical enzymology. There is a good correlation between the methods as shown by $r$ values very close to unity. The comparison method also gave satisfactory results; in almost all instances the slopes were very near unity (except, as mentioned above, for LD, AMS and GGT on the RA-2X, where different procedures were employed), thus excluding the existence of a relevant bias. Also, the study of the systematic error at the clinical decision levels (performance claim) yielded satisfactory results for the Hitachi 737 compared with the other instruments.

### Acknowledgements

This study was supported in part by grants from the Italian National Research Council (CNR) and the Ministry of Public Education (MPI), Rome, Italy.

### References

1. Weisbrot, I. M., in *Statistics for the Clinical Laboratory* (Philadelphia, 1985), p. 129.
2. Committee on Enzymes, Scandinavian Society for Clinical Chemistry and Clinical Physiology, *Scandinavian Journal of Clinical and Laboratory Investigation*, **33** (1974), 291.
3. Committee on Enzymes, Scandinavian Society for Clinical Chemistry and Clinical Physiology, *Scandinavian Journal of Clinical and Laboratory Investigation*, **36** (1976), 711; **39** (1976), 1.
4. Richards, A. H., Lubinsk, R. M. and Vanderlinde, R. E., *Clinical Chemistry*, **21** (1975), 1018.
5. Szasz, G. and Persijn, J. P., *Journal of Clinical Chemistry and Clinical Biochemistry*, **12**, (1974), 228.
6. Committee on Enzymes, Scandinavian Society for Clinical Chemistry and Clinical Physiology, *Scandinavian Journal of Clinical and Laboratory Investigation*, **36** (1976), 119.
7. Hohenwallner, W., Hagele, E. O., Scholer, A. and Staber, G., *Berichte der Österreichischen Gesellschaft für Klinische Chemie*, **6** (1983), 101.
8. Pierre, K. J., Tung, K. K. and Nadj, H., *Clinical Chemistry.*, **22** (1976), 1219.
9. *Proposed Standard PSEP-3, Protocol for Establishing Performance Claims for Clinical Chemical Methods. Replication Experiment* (National Committee for Clinical Laboratory Standards, Villanova, PA, 1979).
10. *Proposed Standard PSEPS-4, Protocol for Establishing Performance Claims for Clinical Chemical Methods. Comparison of Methods Experiment* (National Committee for Clinical Laboratory Standards, Villanova, PA, 1979).
11. *Proposed Standard PSEPS-2, Protocol for Establishing Performance Claims for Clinical Chemical Methods. Introduction and Performance-Check Experiment* (National Committee for Clinical Laboratory Standards, Villanova, PA, 1979).
12. Schwartz, M. K., Statland, B. E. and Coughlin, J., *Clinical Chemistry*, **30** (1984), 364.
13. Statland, B. E. *Clinical Decision Levels for Lab Tests* (Medical Economics Books, Oradel, NJ, 1983), p. 57.
14. Ferrè, C., Castineiras, M. J., Alsina, M. J., Galimany, R. and Riera, J., *Journal of Automatic Chemistry*, **6** (1984), 39.
15. Knedel, M., Haeckel, R. and Seidel, D., *Journal of Clinical Chemistry and Clinical Biochemistry*, **24** (1986), 409.
16. Bais, R., White, R. G., Elston, D. and Geary, T. D., *Journal of Automatic Chemistry*, **4** (1982), 21.

